# Genomic and Proteomic Analyses of the Terminally Redundant Genome of the *Pseudomonas aeruginosa* Phage PaP1: Establishment of Genus PaP1-Like Phages

**DOI:** 10.1371/journal.pone.0062933

**Published:** 2013-05-13

**Authors:** Shuguang Lu, Shuai Le, Yinling Tan, Junmin Zhu, Ming Li, Xiancai Rao, Lingyun Zou, Shu Li, Jing Wang, Xiaolin Jin, Guangtao Huang, Lin Zhang, Xia Zhao, Fuquan Hu

**Affiliations:** Department of Microbiology, College of Basic Medical Science, Third Military Medical University, Chongqing, China; Centro Nacional de Biotecnologia – CSIC, Spain

## Abstract

We isolated and characterized a new *Pseudomonas aeruginosa* myovirus named PaP1. The morphology of this phage was visualized by electron microscopy and its genome sequence and ends were determined. Finally, genomic and proteomic analyses were performed. PaP1 has an icosahedral head with an apex diameter of 68–70 nm and a contractile tail with a length of 138–140 nm. The PaP1 genome is a linear dsDNA molecule containing 91,715 base pairs (bp) with a G+C content of 49.36% and 12 tRNA genes. A strategy to identify the genome ends of PaP1 was designed. The genome has a 1190 bp terminal redundancy. PaP1 has 157 open reading frames (ORFs). Of these, 143 proteins are homologs of known proteins, but only 38 could be functionally identified. Sodium dodecyl sulfate-polyacrylamide gel electrophoresis and high-performance liquid chromatography-mass spectrometry allowed identification of 12 ORFs as structural protein coding genes within the PaP1 genome. Comparative genomic analysis indicated that the *Pseudomonas aeruginosa* phage PaP1, JG004, PAK_P1 and vB_PaeM_C2-10_Ab1 share great similarity. Besides their similar biological characteristics, the phages contain 123 core genes and have very close phylogenetic relationships, which distinguish them from other known phage genera. We therefore propose that these four phages be classified as PaP1-like phages, a new phage genus of *Myoviridae* that infects *Pseudomonas aeruginosa*.

## Introduction

Bacteriophages (phages) are ubiquitous in the biosphere [1]. Estimations of phage numbers, ranging from 10^30^ to 10^32^ in total, are approximately tenfold higher than those of bacteria [2]. Numerous phage investigations have been performed worldwide since Frederick William Twort and Felix dHerelle first reported the discovery of phages in 1915 and 1917, respectively [3], [4]. Phages are potential antimicrobial agents in various clinical or agricultural settings [5], [6] and have become important molecular and biological tools in facilitating the development of bioscience. Approximately 6300 different phages have been examined by electron microscopy [7]; however, only 759 of these (721 infecting bacteria and 38 infecting archaea) have been completely sequenced based on data from the National Center for Biotechnology Information (NCBI; http://www.ncbi.nlm.nih.gov/; Bethesda, MA, USA, Oct. 2012). This number is far lower than the number of sequenced bacteria (3433 complete genomes of bacteria and 199 complete genomes of archaea as of 28 Oct. 2012). A detailed dissection of phage genomes would add valuable data to our knowledge of phages and help us understand the evolutionary relationships between phages and bacteria.

*Pseudomonas aeruginosa* (*P. aeruginosa*), an opportunistic pathogen, is ubiquitous in the environment and often resistant to a large number of antibiotics. As such, the treatment of *P. aeruginosa* infections is very difficult [8]. Investigating the biology of phages is important for humans to fight multiple-drug resistant pathogens [9]. Sixty-three complete genome sequences of *Pseudomonas* phages, most of which infect *P. aeruginosa*, have become available in GenBank as of 28 Oct. 2012. Among these genome sequences, 18 *P. aeruginosa* phages belong to the *Myoviridae* family. Members of this family are efficient killers of bacteria and can affect many aspects of bacterial ecology and evolution. *P. aeruginosa* phages have been studied for decades for use as therapeutics and typing agents [10]. This group of phages seems to be taxonomically diverse and genetically dissimilar [11]. Currently, most characterized myoviruses of *P. aeruginosa* are classified into four genera, namely, phiKZ-like phages, P2-like phages, PB1-like phages, and KPP10-like phages [12]–[15]. Some *P. aeruginosa* phages have been characterized but remain unclassified. Detailed characterizations of novel *P. aeruginosa* phages will be significant for understanding the interactions between *P. aeruginosa* and its phages and the exploration of useful therapeutic reagents against *P. aeruginosa*.

The interests of our group are focused on the dissection of phage genomes and their biological issues. We previously isolated and identified three *P. aeruginosa* phages from the sewages of our affiliated hospitals and designated them as PaP1, PaP2, and PaP3. PaP1 is a virulent phage, whereas both PaP2 and PaP3 are temperate phages. The genome of PaP3 can integrate into the chromosome of the host bacteria through a tRNA gene locus [16].

The present work focuses on the genomic and proteomic analyses of the phage PaP1. The results indicate that PaP1, in taxonomy, belongs to *Myoviridae*, and its genome has terminally redundant ends of 1190 bp. High-performance liquid chromatography-mass spectrometry (HPLC-MS) identified 12 PaP1 structural protein coding genes. Based on comparative genomic analysis, we propose that *P. aeruginosa* phages PaP1, JG004, PAK_P1, and vB_PaeM_C2-10_Ab1 be classified as a new genus named “PaP1-like phages” within the *Myoviridae* family.

## Results

### Morphology of PaP1

**Figure 1:**
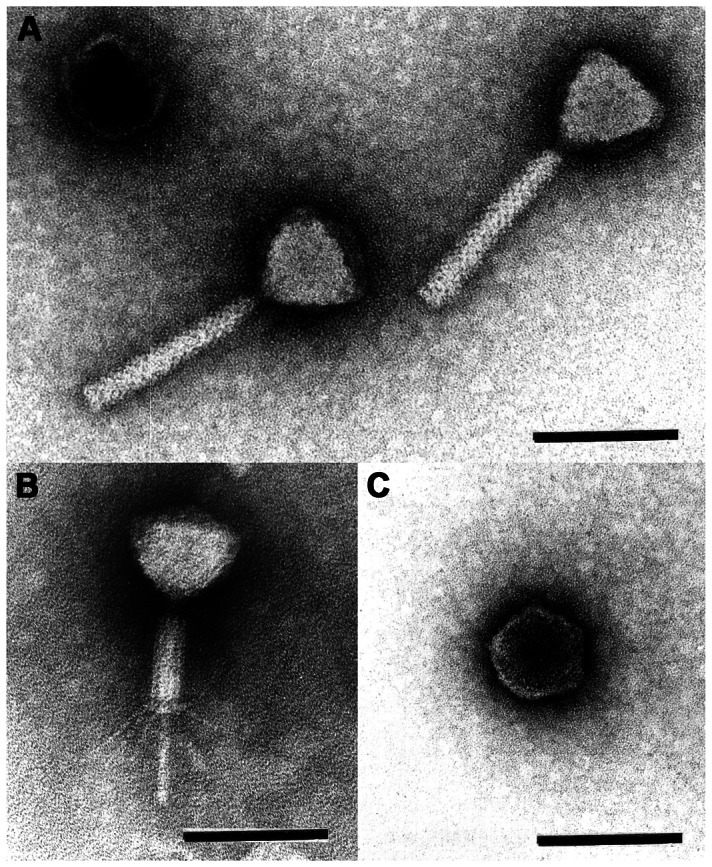
Electron micrographs of purified PaP1 phage particles. **A** shows two PaP1 particles with uncontracted tails and an empty head (uranyl acetate). **B** shows the contracted tail with straight tail fibers (phosphotungstate). **C** shows a pentagonal head (uranyl acetate). The scale bar represents 100 nm. These micrographs were taken by Hans-Wolfgang Ackermann, School of Medicine, Laval University, Quebec, Canada.

PaP1 is in structure and dimensions identical to *P. aeruginosa* phage PB1 [14]. The head is an icosahedron, as evidenced by the simultaneous presence of hexagonal and pentagonal capsids, and measures 68–70 nm between opposite apices (Figure 1). Shallow depressions in uranyl acetate indicate the presence of capsomers. The head is separated from the tail sheath by an 8 nm-long neck. Uncontracted tails measure 138–140 × 17–20 nm. Contracted tails measure 55 × 22 nm. Base plates are poorly visible on extended tails. Upon contraction, they separate from the sheath and appear as disks of 23 × 3 nm. There are at least 4 straight tail fibers (Figure 1B). In the quiescent tail, the fibers are folded along the sheath.

### Biological characteristics of PaP1

**Figure 2:**
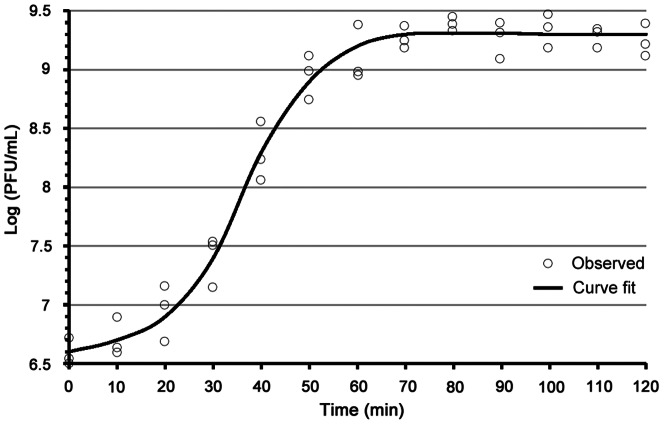
One-step growth curve of phage PaP1. Experiments were repeated three times with duplicate samples. The Y-axis shows the log of plaque forming units per milliliter (PFU/mL).

PaP1 forms clear plaques (∼3 mm in diameter) surrounded by a small semitransparent halo on the lawns of the host bacteria. In rich liquid medium, PaP1 is amplified to high titers (∼10^11^ PFU/ml). PaP1 particles are stable for over two months of storage at 4°C and resistant to chloroform. Based on the one-step growth curve of PaP1 (Figure 2), its latent period is about 20 min, its burst period is about 40 min, and the average number of PaP1 progeny produced from one host bacterium is about 65. PaP1 can lyse two other strains of *P. aeruginosa* (PA4 and PA6), aside from PA1. This result may provide a potential basis for the application of PaP1 in phage therapy.

### General genomic characteristics of PaP1

**Figure 3:**
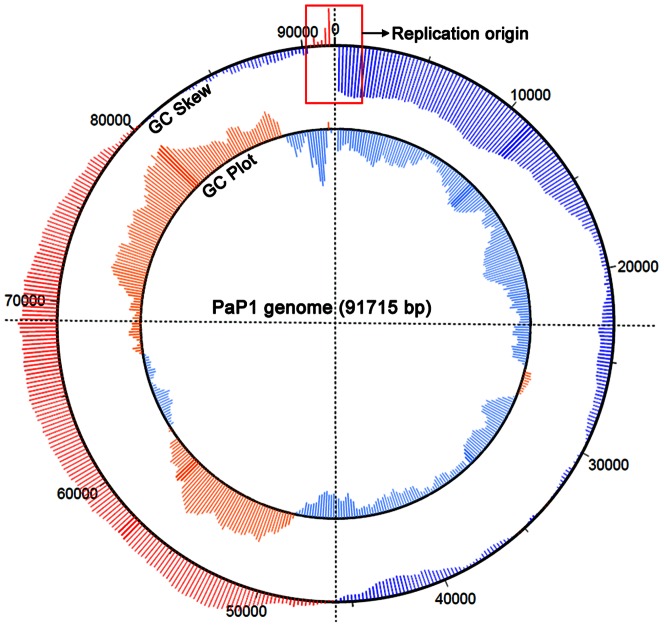
Circular conformation of GC skew and GC plot of the PaP1 genome. The GC skew is calculated as (G-C)/(G+C) and the GC plot shows GC% content plot. The outer circle represents the GC skew (red for positive and blue for negative); the inner circle represents the GC plot (pink for above-average and powder blue for below-average). The red pane indicates the putative replication origin of the PaP1 genome.

The genome of PaP1 consists of 91,715 base pairs (bp) with a G+C content of 49.36%, which is significantly less than that of its host (66.34%). The GC skew of the PaP1 genome is shown in Figure 3. In viral genomes, the lowest point on the GC skew curve is typically the origin of replication [17]. Therefore, the putative replication origin of the PaP1 genome is at the end of the genome sequence (Figure 3).

No direct repeats of more than 50 bp, inverted repeats of more than 26 bp, or mirror repeats of more than 17 bp were found in the PaP1 genome, indicating that it does not contain complicated secondary structures. Twelve tRNA genes were found in the PaP1 genome (Table 1). Among the 12 encoded tRNAs, tRNA^Arg^, tRNA^Gln^, and tRNA^Gly^ are used preferentially by PaP1, but not by the host. Codon usage analysis indicated that the three tRNAs are important for the protein expression of PaP1, since phage tRNA genes can overcome differences in codon usage between the phage and the host [18].

**Table 1:** General features of the PaP1 genome.

Feature	PaP1 genome
Genome size	91,715 bp
G+C content (G+C content host)	49.36% (66.34%)
No. of predicted genes (proteins)	169 (157)
% of the genome with non-coding regions	13.8%
No. of proteins without homologs	14
No. of proteins with determined functions	38
Predicted tRNAs	tRNA^Glu^; tRNA^Phe^; tRNA^Gly^; tRNA^Pro^; tRNA^Asn^; tRNA^Cys^; tRNA^Asp^; tRNA^Ile^; tRNA^Leu^; tRNA^Lys^; tRNA^Arg^; tRNA^Gln^

### Determination of PaP1 genome termini

**Figure 4:**
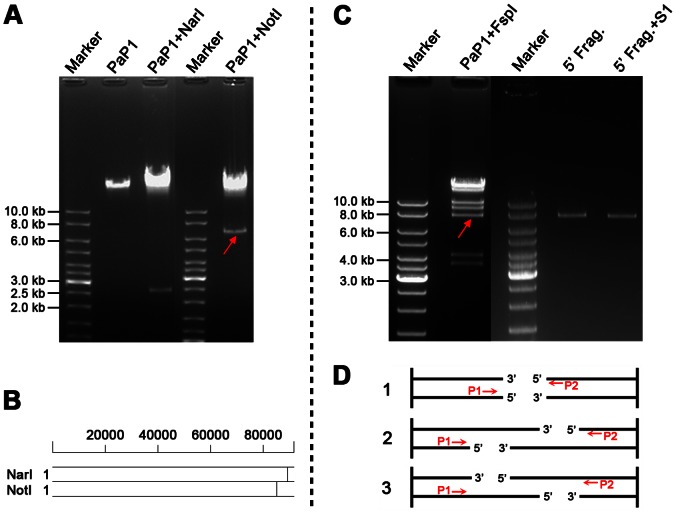
Identification of PaP1 genome ends. **(A)** Digestion of the PaP1 genome DNA by NarI and NotI. The red arrow indicates the 3′ end fragment of the PaP1 genome. **(B)** NarI and NotI restriction map of the PaP1 genome. **(C)** PaP1 DNA was digested by FspI and the recovered 5′ end fragment (5′ Frag., indicated by the red arrow) was digested by S1 nuclease. **(D)** Strategy designed to identify PaP1 genome ends. Primers P1 and P2 are annealed to 3′ and 5′ end fragments of the PaP1 genome DNA, respectively. Terminal run-off sequencing of the two ends (also shown in Figure 5C) is then performed. Case 1 (blunt end): The two sequences obtained by P1 and P2 do not have repeated regions and they can be assembled to the PaP1 genome sequence with no gap between them. Case 2 (3′-protruded end): The two sequences obtained by P1 and P2 also do not have repeated regions; however, a gap is observed between the sequences once assembled to the PaP1 genome sequence. The sequence within the gap is the 3′-protruded cohesive sequence. Case 3 (5′-protruded end or terminal redundancy): A repeat between the two obtained sequences is observed. If the repeated sequence is less than 100 bp, it is regarded as the 5′-protruded cohesive sequence [16], [19], [20]; however, if the repeated sequence is over 100 bp, it is regarded as a terminally redundant sequence [12], [62], [63].

The PaP1 genome was assembled as a circular molecule when sequencing was completed. The restriction endonucleases NarI and NotI, both of which have only one cut site in the genomic DNA, were selected to digest the DNA and released two short bands (about 2.5 and 6.5 kb) in the gel (Figure 4A) This result indicates that the PaP1 genome is a linear molecule. Figure 4B shows the recognition sites of NarI and NotI within the linear PaP1 genome. The DNA band indicated by the red arrow in Figure 4A is the 3′ end fragment of the PaP1 genome. The restriction endonuclease FspI was used to digest the PaP1 genome and release the 5′ end fragment, as indicated by the red arrow in Figure 4C. Both the 3′ and 5′ end fragments were purified and used as templates for terminal run-off sequencing with primers P1 and P2 (Figure 4D), respectively.

**Figure 5:**
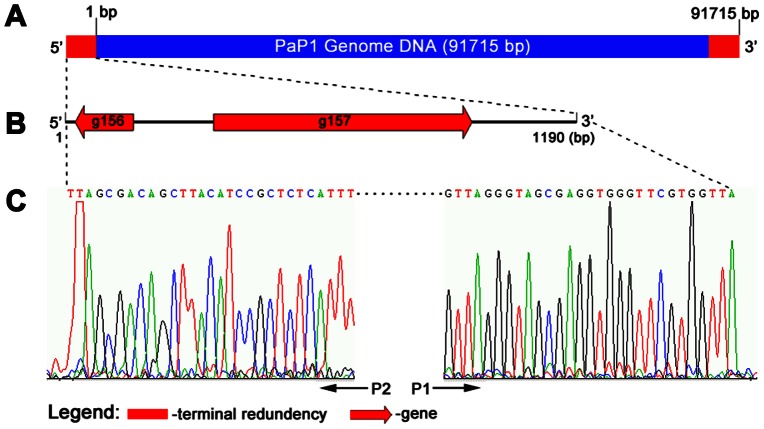
Structure of PaP1 terminal redundancy. **(A)** PaP1 genome DNA with terminal redundancy at both ends. The sequence data of the PaP1 genome are calculated from 1 bp to 91,715 bp. **(B)** Putative genes within the PaP1 terminal redundancy. **(C)** Terminal run-off sequencing chromatograms of both ends of the PaP1 genome. The sequencing direction of primers P1 and P2 is indicated by the black arrows.

The results of terminal run-off sequencing coincided with case 3 in Figure 4D. The two sequences obtained by P1 and P2 have a long repeat of 1190 bp, suggesting that the repeated sequence is terminally redundant rather than cohesive, since the latter is usually less than 100 bp [16], [19], [20]. We used S1 nuclease, a single-strand digesting enzyme, to digest the 5′ end fragment. The cut and uncut 5′ end fragments had the same size (Figure 4C), indicating that the 1190 bp terminal sequence is double-stranded. Thus, the natural structure of the PaP1 genome DNA molecule can be described as shown in Figure 5A. Two functionally unknown genes (g156 and g157) were found in the terminally redundant region (Figure 5B).

### Identification and organization of PaP1 genes

The open reading frames (ORFs) of the PaP1 genome were identified using ORF Finder [21] with ATG, GTG, and TTG as start codons. A total of 541 ORFs (>100 bp) were predicted from the PaP1 genome, among which 157 ORFs were identified as protein coding genes. The average length of a gene is about 504 bp. Only about 13.8% of the PaP1 genome belongs to non-coding regions (Table 1), which is in concordance with the fact that the ORFs of tailed dsDNA phages are tightly and efficiently organized, with little space between genes. The space between genes is usually occupied by putative regulatory sequences, such as promoters and terminators. Putative promoters and terminators of the PaP1 genome are listed in Table 2 and 3, respectively.

**Table 2:** Predicted promoters of the PaP1 genome.

Start	End	Score	Promoter sequence
13877	13922	0.99	TATTTGAGACGAGAGTATTCCATATCTAGGAACATCCCAA**T**AAAAGCTAG
16473	16518	0.99	GGATTGATTGCAACCACCTCGCCTACCAGCATAACCATTC**C**AGTTCCGCC
16814	16859	0.99	AACTTGTGACCGTTACGTTTTACTACTTTCATATTCCCTC**C**TATCGCTTC
19340	19385	0.99	GTCTTTGAAAAACTCACGTTGTGCTTTGTTAAACTTCATT**C**ACTTTTCTC
21533	21578	0.99	CATGTTTTAATACACTTATCAGATCAAGAAATACTTAGCG**A**GTAGCCCTG
22789	22834	1.00	GTCTGATTTGGAATCAGAAGGTCGAAGGTTCAAATCCTTC**C**GGGGTGACC
44680	44725	0.99	TTATAGTGGAATTTAGAAATGTTGTAAAGTATAAAATGGG**T**TACGAGTGC
54108	54153	0.99	GATTCAATTGGAGGTGAACAATGGAGTTGTATGACCAGTG**G**CGTAAGTAT
58145	58190	0.99	AGTTGTGACTCTCAAGGACTACACTCCGGTAGAGTATATT**C**ACCTGCCGC
67819	67864	1.00	CGCTATTTTCAAAATCGACCGCAAATCTAAATAATCGAAA**A**GGAGATATA
68942	68987	0.99	ACTTGTCGAAAGGGAGCAGCTACGATCAGTATAAAAAATC**C**ATGCGGAGC
74233	74278	1.00	GATTTTGTGAATTTGCCTGTGTAACGGAGGATAATCGAGT**A**ATTAGCGTG
75726	75771	1.00	TGGACTTTTAAAGTCAACTAGGAGGAAGTTATGATAAAAG**A**TTTTGTAAA
78179	78224	0.99	ATTATTTTTAGATTTCTGGGAGTTTCGGCCAATATGGGCA**A**TATGGAATT
89416	89461	0.99	AAAGTTGTTGACAGCTTAGGCCATTCCTGTAGAATGGCCA**T**CAAGCAAAG

The score cutoff is 0.99. The transcription start is shown in larger and bolder font.

**Table 3:** Putative terminators of the PaP1 genome.

Terminator name	Chain	Start	Length (bp)	Score
T1	+	450	43	−23.8
T2	−	13972	33	−18.1
T3	+	15874	45	−16.0
T4	+	33220	35	−20.8
T5	+	48630	39	−19.7
T6	+	52853	37	−16.0
T7	+	71138	42	−17.3
T8	+	73693	36	−18.3
T9	+	77027	28	−16.7
T10	+	78134	27	−15.6
T11	+	85403	42	−22.8

The energy threshold value is −15.

**Figure 6:**
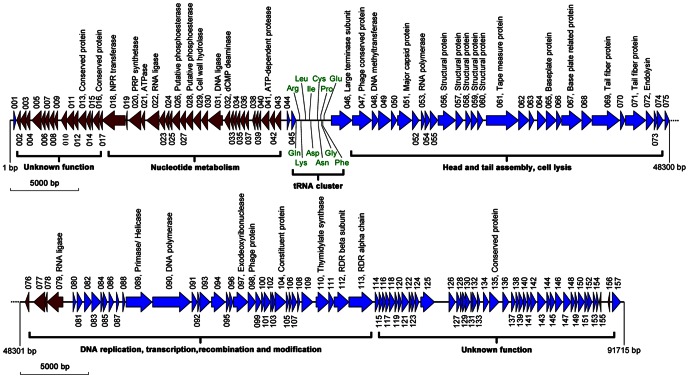
Diagram of the PaP1 genome with functional annotations. Blue arrowheads show genes on the plus strand; brownish red arrowheads show genes on the minus strand; green words refer to the amino acid transferred by the corresponding tRNA. NPR: nicotinamide phosphoribosyl, PRP: phosphoribosylpyrophosphate, RDR: ribonucleotide diphosphate reductase.

Sequence homology analyses revealed putative functions for some of the 157 coded proteins. A total of 143 PaP1 proteins have homologs in other species, only 38 of which are functionally identified. Both the genome sequence of PaP1 and its annotation have been deposited in the GenBank database, under accession number HQ832595. The detailed annotation and organization of the PaP1 genome is listed in Table S1 and illustrated in Figure 6. The data shows that the PaP1 genome may be divided into several functional modules, revealing an apparent mosaic structure, which is one of the striking characteristics of phage genomes. This finding also suggests that tailed phage genomes evolve from combinations of modules from different species [22].

Two functionally unknown modules reside near the 5′ and 3′ ends of the PaP1 genome, respectively. A large number of small genes with unknown functions cluster in the two modules. The 3′ end module consisting of g114 to g157 may play critical roles in early transcriptional events. PaP1 codes for its own RNA and DNA polymerases, which are probably involved in the synthesis of its RNA and DNA molecules. At least 10 genes cluster on the minus strand (Figure 6), which probably controls the nucleotide metabolism system of PaP1. These genes can convert the metabolism of the host cell to produce progeny phages [23]. The products of g032 and g110 are dCMP deaminase and thymidylate synthase, respectively, both of which are involved in dTTP synthesis [24]. Phage-encoded thymidylate synthase appears to have diverged from the precursor of the host bacteria. No sequence homologs to phage integrases, repressors, transposases, or excisionases were found, supporting our conclusion that PaP1 is a lytic phage.

Genes closely related to phage morphogenesis also cluster together, among which 12 genes encoding structural proteins were identified (as below). The products of g029 and g072 are cell wall hydrolase and endolysin, respectively, both of which belong to enzybiotic factors [25], [26]. Although endolysin and holin usually constitute a two-component lysis system for the liberation of phage progenies from the host cell, no holin homolog was found in the PaP1 genome. The endolysin gene (g072) of PaP1 has been expressed in our laboratory, and we have shown that the purified product can hydrolyze the cell wall peptidoglycan of *P. aeruginosa* [27].

### Identification of phage PaP1 structural proteins

**Figure 7:**
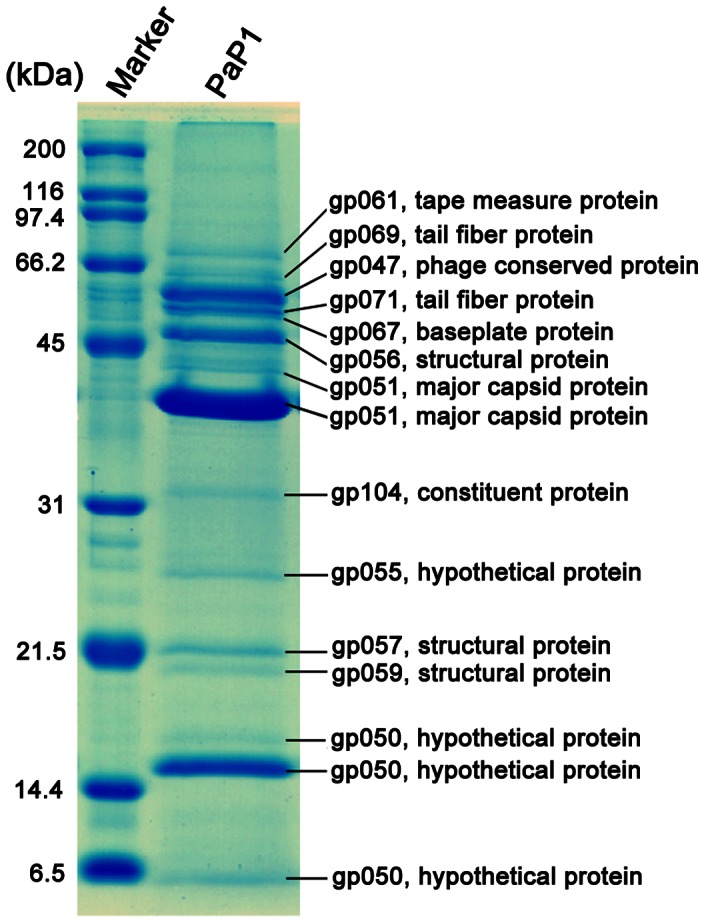
SDS-PAGE analysis of the structural proteins of phage PaP1. Proteins were visualized in a 15% (w/v) gel and identified by HPLC-MS analysis (Table 4).

A dsDNA phage particle is made up of a series of structural proteins and a single DNA molecule containing the entire genome. The PaP1 structural protein coding genes cluster in a module of its genome and are preceded by a terminase gene (Figure 6). The terminase plays an important role in DNA packaging. To identify the structural proteins of PaP1, sodium dodecyl sulfate-polyacrylamide gel electrophoresis (SDS-PAGE) was used to visualize each structural protein in the gel (Figure 7). At least 17 proteins with molecular weights ranging from 6 kDa to 80 kDa were resolved. Each protein band was then excised for HPLC-MS, permitting the allocation of 15 protein bands to 12 corresponding PaP1 genes (Figure 7). The detailed parameters and results of the mass spectrometry are shown in Table 4. The sequence coverage reaches up to 58%. The sequence coverages of gp067 and gp071 are 3% and 4%, respectively, which are relatively low compared with other proteins; hence, the identification of these proteins as structural components of the phage must be confirmed further.

**Table 4:** Mass spectrometry identification of PaP1 structural proteins.

Gene product	MW (kDa)^a^	MW (kDa)^b^	NO. identified peptides	Coverage (%)	Distinct summed MS/MS search score	Mean peptide Spectral intensity
gp061	85.87	80	6	12	105.90	4.35e+007
gp069	69.79	65	7	21	115.79	1.16e+008
gp047	54.23	55	4	13	73.16	1.77e+008
gp071	53.09	53	1	4	12.54	5.17e+007
gp067	52.4	51	1	3	17.57	5.83e+007
gp056	46.37	47	6	18	108.48	2.98e+009
gp051	39.38	43	4	20	58.97	7.36e+008
gp051	39.38	40	11	47	192.95	5.99e+009
gp104	29.81	32	3	17	57.65	2.74e+007
gp055	21.29	25	4	41	77.56	2.05e+007
gp057	18.97	21	5	47	85.63	6.96e+007
gp059	17.70	19	1	13	16.22	6.60e+006
gp050	14.87	16	2	17	34.94	1.60e+007
gp050	14.87	15	6	58	120.18	7.88e+008
gp050	14.87	6	1	15	23.25	2.51e+008

a The MW value is theoretically calculated.

b The MW value is experimentally estimated.

The predominant band is, as predicted, the major capsid protein (gp051, ∼40 kDa); the band (∼43 kDa) just above it was also identified as a major capsid protein by mass spectrometry (Figure 7). Peptides corresponding to gp050 (∼15 kDa) were found in three bands (Figure 7), and similar to gp051, a small band (∼16 kDa) just above it was also identified as gp050. This observation may be explained as the result of the known carry-over effect [28], [29] of the massively overrepresented major capsid protein and gp051 bands. The gp050 band at the bottom of the gel (∼6 kDa) suggests posttranslational processing [30] of gp050. An unusual finding was that gp104 (Figure 7), a p09 homolog of the phage PaP3 (Table S1), is not located in the late gene cluster for phage morphogenesis but among the genes probably involved in DNA replication and control (Figure 6). The four identified PaP1 structural proteins, tape measure, tail fiber, baseplate, and major capsid, with molecular weight ranging from 40 kDa to 80 kDa, are important for phage PaP1 particle formation. Interestingly, the major capsid protein of PaP1 shares molecular weights and amino acid sequences identical to those of the *P. aeruginosa* phages JG004 [31], PAK_P1 [32] and vB_PaeM_C2-10_Ab1 (Table 5), indicating a close relationship among these four phages.

**Table 5:** Comparison of phages with BlastN scores of more than 200 against the PaP1 genome.

Phage	Isolated place	Accession	Length (bp)	BlastN score	E value	Query coverage	Identity	Ref.
JG004	Germany	GU988610	93,017	1.3e^+5^	0.0	94%	96%	[31]
PAK_P1	France	GQ422154	93,398	1.2e^+5^	0.0	96%	95%	[32]
Ab1#	Cote d'Ivoire	HE983845	92,777	1.3e^+5^	0.0	95%	97%	–
KPP10	Japan	AB472900	88,322	2624	0.0	2%	94%	[15]
PAK_P3$	France	HM173082	88,097	2008	2e^−158^	1%	97%	[54]
P3_CHA$	France	HM173081	88,097	2008	2e^−158^	1%	97%	[54]
PaP3	China	AY078382	45,503	441	3e^−48^	<1%	93%	[16]
NH-4	Ireland	JN254800	66,116	224	9e^−53^	<1%	86%	[64]
LMA2	Holland	FM201282	66,530	219	4e^−51^	<1%	85%	[14]
LUZ24	Belgium	AM910650	45,625	301	1e^−31^	<1%	100%	[65]
MR299-2	Ireland	JN254801	44,789	201	1e^−16^	<1%	90%	[64]

# The full name is vB_PaeM_C2-10_Ab1. It was isolated in Cote d'Ivoire:Abidjan by Christiane Essoh.

$ The genome sequences of PAK_P3 and P3_CHA are almost identical with only two single nucleotide mutations [54]. We chose the genome sequence of PAK_P3 (with more detailed annotations) for subsequent analysis.

### Comparative genomic analysis of PaP1, JG004, PAK_P1, and vB_PaeM_C2-10_Ab1

#### Comparative analysis of genomes

Many PaP1 proteins share great similarity with the homologs of the *P. aeruginosa* phages PAK_P1, JG004, and vB_PaeM_C2-10_Ab1, among which 15 PaP1 proteins share 100% sequence identity with PAK_P1 homologs (Table S1). When the PaP1 genome sequence was compared with the nucleotide database, 11 *P. aeruginosa* phages were found with BlastN scores of more than 200 (Table 5). In particular, PAK_P1, JG004, and vB_PaeM_C2-10_Ab1 showed query coverages of more than 90% (Table 5).

**Figure 8:**
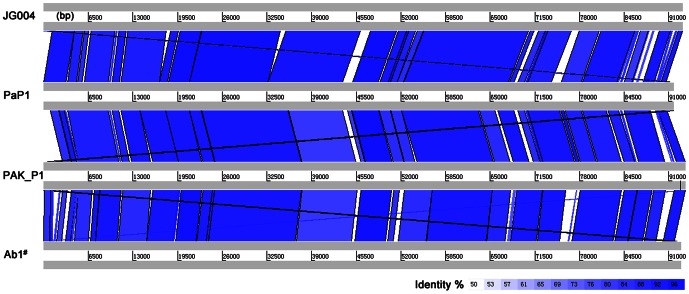
Pairwise nucleotide sequence comparison of phages closely related to PaP1. Comparisons were conducted using BLAST 2.25 and displayed using ACT [56]. Highly related sequences are shown by the blue shadings. The intensity of the blue coloration indicates the level of sequence similarity. The minimum score cutoff is 100 and the minimum identity cutoff is 50%. ^#^The full name is vB_PaeM_C2-10_Ab1.

**Figure 9:**
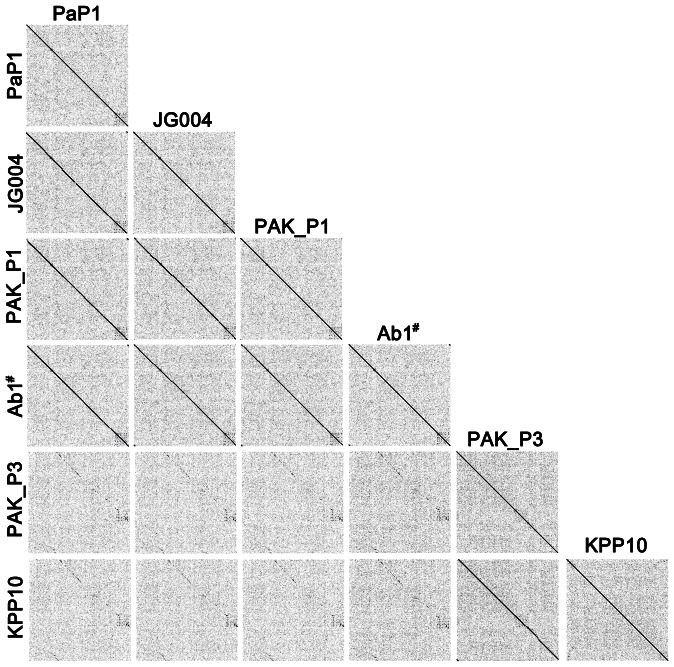
Dot plot of genome sequences of six phages using the program Gepard. The word length used is 9 bp; other parameters are set by default. The black dots indicate that the corresponding genome regions of the abscissa and the ordinate show similarity to each other. ^#^The full name is vB_PaeM_C2-10_Ab1.

A graphical comparison (Figure 8) was performed to illustrate the genomic similarities of PaP1, JG004, PAK_P1, and vB_PaeM_C2-10_Ab1. This figure is a visualized description of the corresponding data listed in Table 5. The whole genome sequences of phage PaP1, JG004, PAK_P1, and vB_PaeM_C2-10_Ab1 show great similarities and most of their DNA sequences appear to have descended from a single common ancestral phage. We also performed a dot plot comparison of the genome sequences of PaP1, JG004, PAK_P1, vB_PaeM_C2-10_Ab1, PAK_P3, and KPP10 (Figure 9). The results are in concordance with Table 5 and Figure 8.

At the protein level, PaP1, JG004, PAK_P1, and vB_PaeM_C2-10_Ab1 show striking similarities. Intriguingly, the major capsid proteins of these four phages share 100% sequence identity with each other, which explains their similar morphologies [31,32, and the present work]. As shown in Figure 8, the regions with no blue shading represent minor insertions or deletions among the genome sequences of these four phages. These DNA regions are probably the accumulated mutations for phage adaptation to the host bacteria. The main differences between these four phage genomes are located in their tail fiber encoding genes, indicating that these phages may have evolved different host cell adsorption mechanisms.

#### Protein homology analysis

The genomic comparison indicated that phages PaP1, JG004, PAK_P1, and vB_PaeM_C2-10_Ab1 are closely related. To investigate this further, protein homology analysis was performed. The result reveals that the PaP1 genome shares 123 (78.34%) homologs with JG004, PAK_P1, and vB_PaeM_C2-10_Ab1, and shares 55 (35.03%) homologs with KPP10 and PAK_P3, and shares even fewer homologs with other phage groups. These results strongly indicate that PaP1, JG004, PAK_P1, and vB_PaeM_C2-10_Ab1 are closely associated and distinguishable from other phage groups. Therefore, these four phages do not belong to any known phage genus and may have descended from a common ancestor.

#### Phylogenetic analysis

**Figure 10:**
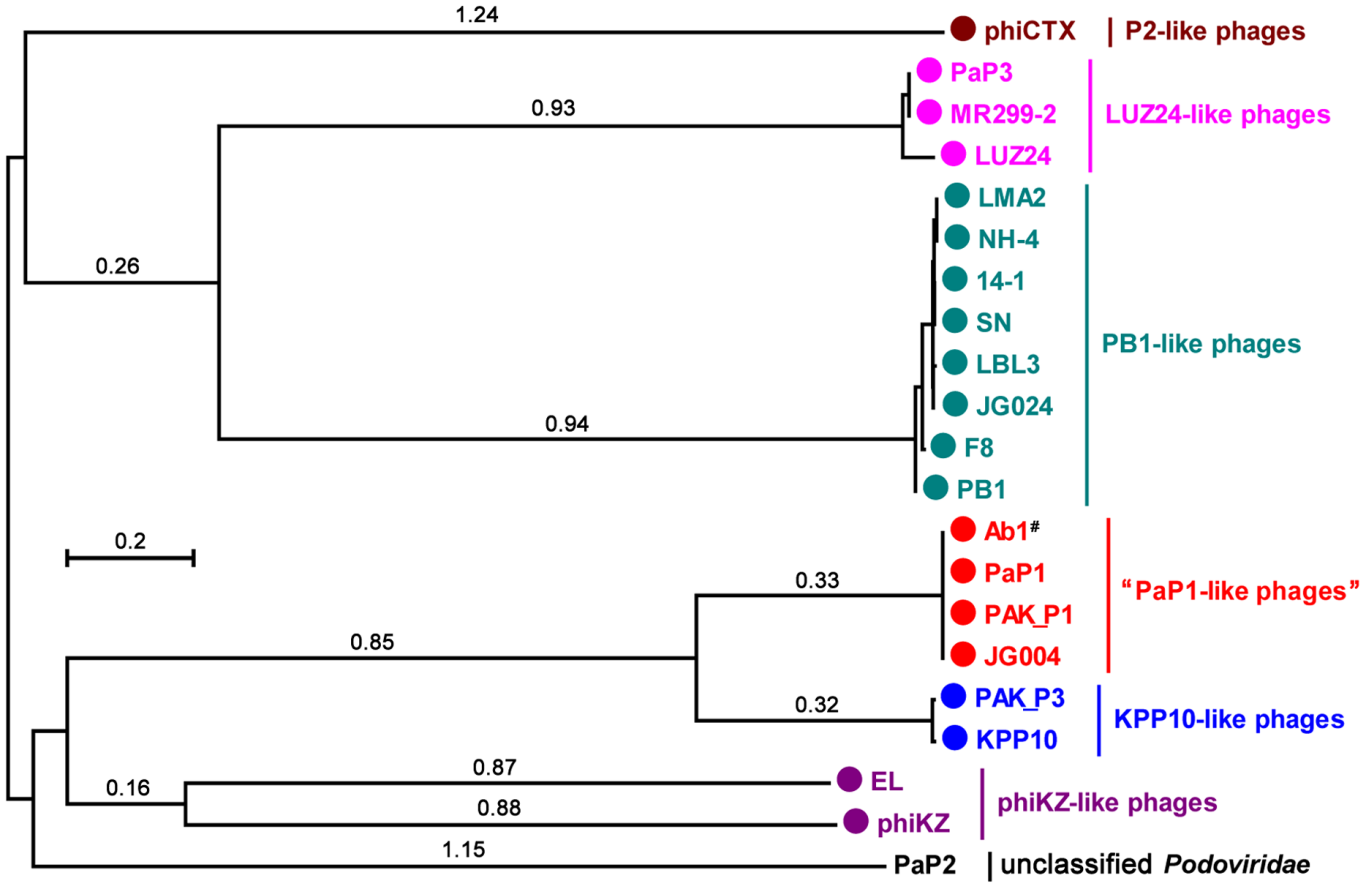
Phylogenetic analysis of major capsid protein amino acid sequences. The diagram was constructed using the MEGA5 program [58]. The relative distances of each main branch are shown in the figure. Both PaP2 and LUZ24-like phages belong to the family of *Podoviridae*; all other phage groups belong to the *Myoviridae* phage family. The phages in the same group are marked with the same color. The group of “PaP1-like phages” is first presented in this work. ^#^The full name is vB_PaeM_C2-10_Ab1.

Phylogenetic analysis was performed based on the major capsid proteins. Since related phages are considered to have similar head structural components, they may be clustered based on their major capsid proteins [33]. We chose phages that are listed in Tables 5 and 6 to analyze the phylogenetic relationships between them, and a phylogenetic tree was constructed (Figure 10). Phage PaP2 was included in the tree because it was identified from the same sample (hospital sewage) from which PaP1 and PaP3 were obtained.

**Table 6:** Overview of genomic characteristics of *Myoviridae* that infect *P. aeruginosa*.

Genus	Phage	Isolated place	Accession	Genome size (bp)	Protein (n)	tRNAs (n)	% GC	Genome ends	Ref.
phiKZ-like phages	phiKZ	Belgium	AF399011	280,334	306	6	36.8	TR	[12]
	EL	Belgium	AJ697969	211,215	201	1	49.3	TR	[66]
P2-like phages	phiCTX	Japan	AB008550	35,580	47	–	62.6	5′ cos	[13]
PB1-like phages	PB1	Scotland	EU716414	65,764	93	–	54.9	NP	[14]
	SN	Russia	FM887021	66,390	92	–	55.6	NP	[14]
	14-1	Germany	FM897211	66,235	90	–	55.6	NP	[14]
	LMA2	Holland	FM201282	66,530	94	–	55.6	NP	[14]
	LBL3	Spain	FM201281	64,427	88	–	55.5	NP	[14]
	F8	Canada	DQ163917	66,015	91	–	54.9	NP	[11]
	JG024	Germany	GU815091	66,275	94	–	55.6	NP	[41]
	NH-4	Ireland	JN254800	66,116	94	–	55.5	NP	[64]
KPP10-like phages	KPP10	Japan	AB472900	88,322	146	3	54.8	TR	[15]
	P3_CHA	France	HM173081	88,097	185	2	54.8	NR	[54]
	PAK_P3	France	HM173082	88,097	185	2	54.8	NR	[54]
Unclassified *Myoviridae*	PaP1	China	HQ832595	91,715	157	12	49.4	TR	PW
	PAK_P1	France	GQ422154	93,398	158	12	49.5	NR	[32]
	JG004	Germany	GU988610	93,017	161	12	49.3	TR	[31]
	Ab1#	Cote d'Ivoire	HE983845	92,777	158	11	49.3	NR	–

Data listed in the table are mainly obtained from the public genome sequences as of 28 Oct. 2012.

TR: Terminally redundant, NP: Non-permuted, cos: Cohesive ends, NR: Not reported, PW: Present work.

# The full name is vB_PaeM_C2-10_Ab1.

Figure 10 shows that different *P. aeruginosa* phage genera cluster in the phylogenetic tree based on the major capsid proteins. This observation is in accordance with the data listed in Table 6, in which myoviruses of *P. aeruginosa* have been assigned to several phage genera, except for PaP1, JG004, PAK_P1, and vB_PaeM_C2-10_Ab1. As expected, these four phages are closely clustered, distinguishing them from other *P. aeruginosa* phages. This finding reinforces the idea that these four phages descend from a common ancestor. Thus, PaP1, JG004, PAK_P1, and vB_PaeM_C2-10_Ab1 should be grouped as a new phage genus: “PaP1-like phages” (Figure 10).

## Discussion

In the present work, the newly isolated phage PaP1 was assigned as a member of the *Myoviridae* family. Over 96% of the investigated phages belong to the tailed phages. A total of 6054 tailed phages are known, among which 1558 are from the family *Myoviridae* [7]. Although members of *Myoviridae* have many common features, they actually represent a diverse collection of phages.

Phage PaP1 has a linear genome consisting of 91,715 bp with a terminal redundancy of 1190 bp. We designed a new strategy to determine the genome ends of PaP1 (Figure 4D) and this strategy is useful for the identification of genome ends of many other phages. Many known phages have terminally redundant genomes, such as phages T3, T7, P22, SPP1, and T4. Similar to PaP1, several *Myoviridae* phages of *P. aeruginosa* (e.g., phiKZ, EL, JG004, and KPP10) also have terminal redundancies (Table 6). These phages employ a variety of mechanisms to generate long DNA concatemers with terminal redundancy, which ensures phage replication without any loss of genetic information [34]. Intriguingly, the terminally redundant region of phage SPO1 contains a “host take-over module” composed of a cluster of 24 genes. This region is responsible for shutting off transcription and translation of the host genes [35]. The terminally redundant region of the PaP1 genome contains two genes with unknown functions (Figure 5B). Identification of the two genes, the PaP1 terminase, and the terminase recognition (pac) site [36], may provide a basis for a understanding of phage PaP1 morphogenesis.

The 91,715 bp PaP1 genome encodes 157 putative proteins. Based on the predicted functions of these proteins, the genome can be divided into several functional modules, showing an apparent mosaic structure that is characteristic of the phage genomes [22], [37]. The PaP1 genome also contains 12,660 bp worth of non-coding regions. Non-coding cannot be interpreted as an indicator of no biological function because some ncRNAs (non-coding RNAs) or DNA binding motifs may be observed within the non-coding regions in phage genomes, which may be useful for the phages but toxic to their hosts [38]. Less than a quarter of the 157 putative proteins have homologs with known functions. Twelve ORFs of the PaP1 genome were identified as structural protein coding genes (Figure 7). The majority of the phage ORFs have unknown functions, which hinders phage studies. The sequence data of these 12 structural proteins have been added to the phage proteomic pool. As similar data emerge, the collective information will be valuable for future phage studies.

Comparative genome analysis revealed that the PaP1 genome shows great similarity with JG004, PAK_P1, and vB_PaeM_C2-10_Ab1 both at the DNA and protein levels, distinguishing them from other *Myoviridae*. In essence, the phages have very few relationships with other phage genera. Some similarities consistently exist among the tailed phages, suggesting that phages may undergo genetic material exchange from a large shared pool [22]. Substantial evidence suggests that tailed phages may be of very ancient origin and it has been proposed that all of the dsDNA tailed phages share common ancestry [39]. The comparison of coat protein structure and virion architecture can provide a sound basis for grouping viruses together [33]. The major capsid protein of PaP1 shares 100% identity with those of JG004, PAK_P1, and vB_PaeM_C2-10_Ab1; their particles also share identical morphology. This observation supports the idea that PaP1, JG004, PAK_P1, and vB_PaeM_C2-10_Ab1 can be grouped together, as shown in Figure 10.

According to similarities in biological characteristics and both DNA and protein sequences, prominent core genes, and the close phylogenetic relationships among PaP1, JG004, PAK_P1, and vB_PaeM_C2-10_Ab1, we propose that these four phages, with PaP1 as the type virus, can be grouped as a new genus (PaP1-like phages) of myovirus bacteriophages, as shown in Figure 10. We predict that other newly characterized phages will be assigned to “PaP1-like genus” in the near future, thereby contributing to our understanding of phage biology.

## Materials and Methods

### Pseudomonas aeruginosa strains

Six *P. aeruginosa* strains (PA1, PA2, PA3, PA4, PA5, and PA6) were isolated at the second affiliated hospital of the Third Military Medical University, Chongqing, China, and cultivated in our laboratory. These strains belong to serogroups 9, 20, 6, 6, 20, and 11 of the *P. aeruginosa* international antigenic typing system, respectively. All six strains were cultivated at 37°C in LB medium with shaking for ∼5 h to reach the log phase.

### Phage propagation and purification

Phage PaP1 was isolated from the hospital sewage using *P. aeruginosa* PA1 as host bacterium, based on a standard lambda phage isolation protocol [40]. A liquid culture of the PA1 strain of the log phase growth was infected with PaP1 (MOI of 1/100) and incubated at 37°C with shaking. After ∼5 h, the culture showed signs of lysis, and a few drops of chloroform were added to it. The culture was then centrifuged at 10,000 *g* for 5 min, and the supernatant was stored at 4°C for subsequent experiments. After storage at 4°C for over two months, the supernatant was diluted, plated onto a Petri dish overlaid with the PA1 stain, and then cultured at 37°C until individual plaques could be picked to test the titers of PaP1 in the supernatant. Another 5 *P. aeruginosa* strains (PA2, PA3, PA4, PA5, and PA6) were used as host bacteria to test whether or not PaP1 could lyse them. One-step growth experiments of PaP1 were performed, as previously described [41], to determine phage growth characteristics. Crude phage suspensions of PaP1 were concentrated and purified by PEG8000 precipitation according to the method of Govind et al. [42]. The purified PaP1 particles were further purified using CsCl gradient ultracentrifugation [43].

### Transmission electron microscopy (TEM)

Filtered phage lysates (about 10^11^ PFU/mL) were sedimented for 60 min at 25,000 *g* in a Beckman J2-21 centrifuge (Palo Alto, CA, USA) equipped with a JA1.1 fixed-angle rotor, followed by washing in neutral ammonium acetate buffer (0.1 M) under the same conditions. Phage particles were deposited on carbon-coated copper grids, stained with uranyl acetate (2%, pH 4.5) or potassium phosphotungstate (2%, pH 7.0), and examined under a Philips EM 300 electron microscope. Magnification was monitored with T4 phage tails. Dimensions of PaP1 particles are calculated from 20 particles.

### DNA extraction and sequencing

EDTA to a final concentration of 20 mM, proteinase K at 50 µg mL^−1^, and sodium dodecyl sulfate at 0.5% (w/v) were added to the purified phage PaP1 stock solution. The mixture was incubated at 56°C for 1 h, after which an equal volume of phenol-chloroform-isoamyl alcohol (25∶24∶1) was added to it, followed by centrifugation at 5000 *g* for 10 min. The aqueous layer was extracted with chloroform at 5000 *g* for 10 min. The aqueous layer was collected, mixed with 0.6 volumes of isopropanol, and then stored at −20°C for one night. The mixture was centrifuged at 4°C and 12,000 *g* for 10 min, and the precipitated DNA was collected and washed with 70% and 100% ethanol, respectively. The obtained PaP1 DNA was suspended in TE buffer (pH 8.0) and stored at −20°C for use. DNA sequencing was carried out at the Chinese National Human Genome Center (Shanghai, China) using the Roche/454 GS FLX Titanium system [44]. Roche/454 sequence reads were assembled using the Phred/Phrap/Consed software package [45].

### Analysis of PaP1 genome ends

Simulation of the restriction enzyme mapping of the PaP1 genome sequence was performed using the software package DNAStar [46]. The PaP1 DNA was digested by selected restriction endonucleases (NarI, NotI, and FspI, purchased from New England Biolabs, Ipswich, MA, USA). For a reaction system of 20 µL, 10 units of the restriction endonuclease (NarI or NotI) and 200 ng of PaP1 DNA were used. The mixture was incubated at 37°C for 120 min and then used to perform agarose gel electrophoresis. For a reaction system of 100 µL, 1 µg of PaP1 genome DNA and 50 units of restriction endonuclease (NotI or FspI) were used. The mixture was incubated at 37°C for 100 min. Agarose gel electrophoresis was subsequently performed to separate the restriction fragments containing the 5′ and 3′ ends of the PaP1 genome. The restriction fragments containing the 5′ and 3′ ends of the PaP1 genome were purified using Wizard SV Gel and PCR Clean-up System (Promega, Fitchburg, WI, USA), respectively. Terminal run-off sequencing was carried out by BGI-Shenzhen (Shenzhen, China). The 3′ end fragment was sequenced using primer P1 (5′-CGTTCGACGATCCGATGC-3′), and terminal run-off sequencing of the 3′ end fragment was performed by P1. The 5′ end fragment was sequenced using primer P2, which represents three primers (P2a, P2b, and P2c). P2a (5′-CGCCGATGGTCTAGCTGTTG-3′) was the first primer used to sequence the 5′ end fragment. P2b (5′-TCGCCTTCTGCCAGTTATG-3′) was designed based on the DNA sequence acquired by P2a. P2c (5′-ATGCCTTGTCGCAGTTGG-3′) was designed based on the DNA sequence acquired by P2b, and terminal run-off sequencing of the 5′ end fragment was performed by P2c. These primers were prepared by BGI-Shenzhen (Shenzhen, China). We used a strategy to explore the terminal sequence of the PaP1 DNA (Figure 4D). Digestion of the 5′ end fragment with S1 nuclease (Takara Bio, Shiga, Japan) at 23°C for 20 min was carried out to further identify the terminally redundant genome of phage PaP1.

### Sequence analysis and genome annotation

The software packages DNAStar [46] and DNAMAN (http://www.lynnon.com/) were used to analyze the basic features of the PaP1 genome sequence. The GC skew of the PaP1 genome was analyzed using DNAPlotter [47]. The internet tool tRNAscan-SE 1.21 [48] was used to predict tRNA genes in the DNA sequence with a cove score cutoff of 20. ORFs were analyzed using NCBI ORF Finder [21], and phage genes were predicted using the software GeneMark.HMM [49] with a length threshold of 100 bp. DNA sequences and protein sequences were scanned for homologs using BLAST [50]. Predicted promoter regions were identified using neural network promoter prediction [51], and putative terminator structures were identified using the web tool FindTerm (http://linux1.softberry.com/berry.phtml).

### SDS-PAGE and HPLC-MS of the PaP1 structural proteins

The purified phage particles were resuspended in SDS-PAGE loading buffer [52] and boiled for 5 min before loading onto a 15% (w/v) polyacrylamide gel to identify structural proteins of phage PaP1. We also performed 12% (w/v) and 10% (w/v) SDS-PAGE to better separate proteins of different molecular weight ranges. Protein bands were visualized by staining with Coomassie Brilliant Blue R250 dye for 1 h with shaking and washing with methanol-acetic acid-H_2_O (5∶1∶4). Slices were excised from the gel and digested as described previously [53]. HPLC-MS was performed using an HPLC-CHIP-MS/MS ION TRAP 6330 system (Agilent, Santa Clara, CA, USA). The acquired data (mass signals) were compared with all of the putative protein sequences of PaP1 using Mill proteomics software (Rev A.03.02.060; Agilent, Santa Clara, CA, USA) to determine genes with products corresponding to the selected protein bands.

### Comparative genome analysis

Sequences of the *P. aeruginosa* phages JG004 [31], vB_PaeM_C2-10_Ab1 (GenBank accession No.: HE983845), PAK_P1 [32], KPP10 [15], and PAK_P3 [54] were compared with PaP1. Dot plot comparison of the phage genomes was performed and displayed using Gepard [55]. Paired genome comparison was conducted using BLAST [50] and the results were displayed using ACT [56]. Multiple sequence alignments of major head proteins (also called major capsid proteins) were conducted using ClustalW [57] with default parameters. A phylogenetic tree was also constructed and displayed using MEGA5 [58] via the neighbor-joining method [59]. Protein homology analysis of PaP1, JG004, PAK_P1, and vB_PaeM_C2-10_Ab1 was performed by CoreGenes [60], [61] with a BlastP threshold score of 100.

## Supporting Information

Table S1 **Predicted genes and proteins of phage PaP1.**
(DOCX)

## References

[1] Lima-Mendez G, Toussaint A, Leplae R (2007) Analysis of the phage sequence space: the benefit of structured information. Virology 365: 241–249. 17482656 10.1016/j.virol.2007.03.047

[2] Hendrix RW (2003) Bacteriophage genomics. Curr Opin Microbiol 6: 506–511. 14572544 10.1016/j.mib.2003.09.004

[3] Twort A (1993) In focus, out of step: a biography of Frederick William Twort F.R.S., 1877–1950. Phoenix Mill; Dover, NH: A. Sutton. xi, 40 p.

[4] dHerelle F (1917) Sur un microbe invisible antagoniste des bacilles dysentériques. C R Acad Sci Paris 165: 373–375.

[5] Debarbieux L (2008) Experimental phage therapy in the beginning of the 21st century. Med Mal Infect 38: 421–425. 18692973 10.1016/j.medmal.2008.06.014

[6] Abedon S (2011) Phage therapy pharmacology: calculating phage dosing. Adv Appl Microbiol 77: 1–40. 22050820 10.1016/B978-0-12-387044-5.00001-7

[7] Ackermann HW, Prangishvili D (2012) Prokaryote viruses studied by electron microscopy. Arch Virol 157: 1843–1849. 22752841 10.1007/s00705-012-1383-y

[8] Bonomo RA, Szabo D (2006) Mechanisms of multidrug resistance in Acinetobacter species and *Pseudomonas aeruginosa* . Clin Infect Dis 43 Suppl 2S49–56. 16894515 10.1086/504477

[9] Skurnik M, Strauch E (2006) Phage therapy: facts and fiction. Int J Med Microbiol 296: 5–14. 10.1016/j.ijmm.2005.09.002 16423684

[10] Ceyssens PJ, Lavigne R (2010) Bacteriophages of *Pseudomonas* . Future Microbiol 5: 1041–1055. 20632804 10.2217/fmb.10.66

[11] Kwan T, Liu J, Dubow M, Gros P, Pelletier J (2006) Comparative genomic analysis of 18 *Pseudomonas aeruginosa* bacteriophages. J Bacteriol 188: 1184–1187. 16428425 10.1128/JB.188.3.1184-1187.2006 PMC1347338

[12] Mesyanzhinov VV, Robben J, Grymonprez B, Kostyuchenko VA, Bourkaltseva MV, et al (2002) The genome of bacteriophage phiKZ of *Pseudomonas aeruginosa* . J Mol Biol 317: 1–19. 11916376 10.1006/jmbi.2001.5396

[13] Nakayama K, Kanaya S, Ohnishi M, Terawaki Y, Hayashi T (1999) The complete nucleotide sequence of phi CTX, a cytotoxin-converting phage of *Pseudomonas aeruginosa*: implications for phage evolution and horizontal gene transfer via bacteriophages. Mol Microbiol 31: 399–419. 10027959 10.1046/j.1365-2958.1999.01158.x

[14] Ceyssens PJ, Miroshnikov K, Mattheus W, Krylov V, Robben J, et al (2009) Comparative analysis of the widespread and conserved PB1-like viruses infecting *Pseudomonas aeruginosa* . Environ Microbiol 11: 2874–2883. 19678828 10.1111/j.1462-2920.2009.02030.x

[15] Uchiyama J, Rashel M, Takemura I, Kato S, Ujihara T, et al (2012) Genetic characterization of *Pseudomonas aeruginosa* bacteriophage KPP10. Arch Virol 157: 733–738. 22218962 10.1007/s00705-011-1210-x

[16] Tan Y, Zhang K, Rao X, Jin X, Huang J, et al (2007) Whole genome sequencing of a novel temperate bacteriophage of *P. aeruginosa*: evidence of tRNA gene mediating integration of the phage genome into the host bacterial chromosome. Cell Microbiol 9: 479–491. 16965514 10.1111/j.1462-5822.2006.00804.x

[17] Grigoriev A (1999) Strand-specific compositional asymmetries in double-stranded DNA viruses. Virus Res 60: 1–19. 10225270 10.1016/s0168-1702(98)00139-7

[18] Diard M, Garry L, Selva M, Mosser T, Denamur E, et al (2010) Pathogenicity-associated islands in extraintestinal pathogenic *Escherichia coli* are fitness elements involved in intestinal colonization. J Bacteriol 192: 4885–4893. 20656906 10.1128/JB.00804-10 PMC2944530

[19] Wu R, Taylor E (1971) Nucleotide sequence analysis of DNA. II. Complete nucleotide sequence of the cohesive ends of bacteriophage lambda DNA. J Mol Biol 57: 491–511. 4931680 10.1016/0022-2836(71)90105-7

[20] Bowden DW, Modrich P (1985) In vitro maturation of circular bacteriophage P2 DNA. Purification of ter components and characterization of the reaction. J Biol Chem 260: 6999–7007. 2987239

[21] Wheeler DL, Church DM, Federhen S, Lash AE, Madden TL, et al (2003) Database resources of the National Center for Biotechnology. Nucleic Acids Res 31: 28–33. 12519941 10.1093/nar/gkg033 PMC165480

[22] Hendrix RW, Smith MC, Burns RN, Ford ME, Hatfull GF (1999) Evolutionary relationships among diverse bacteriophages and prophages: all the world's a phage. Proc Natl Acad Sci U S A 96: 2192–2197. 10051617 10.1073/pnas.96.5.2192 PMC26759

[23] Pajunen MI, Kiljunen SJ, Soderholm ME, Skurnik M (2001) Complete genomic sequence of the lytic bacteriophage phiYeO3-12 of *Yersinia enterocolitica* serotype O:3. J Bacteriol 183: 1928–1937. 11222590 10.1128/JB.183.6.1928-1937.2001 PMC95087

[24] Zhang Y, Maley F, Maley GF, Duncan G, Dunigan DD, et al (2007) Chloroviruses encode a bifunctional dCMP-dCTP deaminase that produces two key intermediates in dTTP formation. J Virol 81: 7662–7671. 17475641 10.1128/JVI.00186-07 PMC1933376

[25] Manoharadas S, Witte A, Blasi U (2009) Antimicrobial activity of a chimeric enzybiotic towards *Staphylococcus aureus* . J Biotechnol 139: 118–123. 18940209 10.1016/j.jbiotec.2008.09.003

[26] Wu H, Lu H, Huang J, Li G, Huang Q (2012) EnzyBase: a novel database for enzybiotic studies. BMC Microbiol 12: 54. 22489867 10.1186/1471-2180-12-54 PMC3364899

[27] Sun WZ, Tan YL, Jia M, Hu XM, Rao XC, et al (2010) Functional characterization of the endolysin gene encoded by *Pseudomonas aeruginosa* bacteriophage PaP1. African Journal of Microbiology Research 4: 933–939.

[28] Williams EA, Degnan SM (2009) Carry-over effect of larval settlement cue on postlarval gene expression in the marine gastropod *Haliotis asinina* . Mol Ecol 18: 4434–4449. 19793199 10.1111/j.1365-294X.2009.04371.x

[29] Ciprandi G, Sormani MP, Filaci G, Fenoglio D (2008) Carry-over effect on IFN-gamma production induced by allergen-specific immunotherapy. Int Immunopharmacol 8: 1622–1625. 18691675 10.1016/j.intimp.2008.07.007

[30] Eyer L, Pantucek R, Zdrahal Z, Konecna H, Kasparek P, et al (2007) Structural protein analysis of the polyvalent *Staphylococcal* bacteriophage 812. Proteomics 7: 64–72. 10.1002/pmic.200600280 17154272

[31] Garbe J, Bunk B, Rohde M, Schobert M (2011) Sequencing and characterization of *Pseudomonas aeruginosa* phage JG004. BMC Microbiol 11: 102. 21569567 10.1186/1471-2180-11-102 PMC3120641

[32] Debarbieux L, Leduc D, Maura D, Morello E, Criscuolo A, et al (2010) Bacteriophages Can Treat and Prevent *Pseudomonas aeruginosa* Lung Infections. Journal of Infectious Diseases 201: 1096–1104. 20196657 10.1086/651135

[33] Bamford DH, Grimes JM, Stuart DI (2005) What does structure tell us about virus evolution? Curr Opin Struct Biol 15: 655–663. 16271469 10.1016/j.sbi.2005.10.012

[34] Keppel F, Fayet O, Georgopoulos C (1988) Strategies of bacteriophage DNA replication. New York: Plenum Press. 145–262 p.

[35] Stewart CR, Gaslightwala I, Hinata K, Krolikowski KA, Needleman DS, et al (1998) Genes and regulatory sites of the “host-takeover module” in the terminal redundancy of *Bacillus subtilis* bacteriophage SPO1. Virology 246: 329–340. 9657951 10.1006/viro.1998.9197

[36] Bravo A, Alonso JC, Trautner TA (1990) Functional analysis of the *Bacillus subtilis* bacteriophage SPP1 pac site. Nucleic Acids Res 18: 2881–2886. 2161515 10.1093/nar/18.10.2881 PMC330814

[37] Casjens S, Hatfull G, Hendrix R (1992) Evolution of dsDNA tailed-bacteriophage genomes. Semin Virol 3: 383–397.

[38] Kimelman A, Levy A, Sberro H, Kidron S, Leavitt A, et al (2012) A vast collection of microbial genes that are toxic to bacteria. Genome Res 22: 802–809. 22300632 10.1101/gr.133850.111 PMC3317161

[39] Monod C, Repoila F, Kutateladze M, Tetart F, Krisch HM (1997) The genome of the pseudo T-even bacteriophages, a diverse group that resembles T4. J Mol Biol 267: 237–249. 9096222 10.1006/jmbi.1996.0867

[40] Sambrook J, Russel DW (2001) Molecular Cloning: A Laboratory Manual, 3rd ed. New York: Cold Spring Harbor Laboratory Press. 170 p.

[41] Garbe J, Wesche A, Bunk B, Kazmierczak M, Selezska K, et al (2010) Characterization of JG024, a *Pseudomonas aeruginosa* PB1-like broad host range phage under simulated infection conditions. BMC Microbiol 10: 301. 21110836 10.1186/1471-2180-10-301 PMC3008698

[42] Govind R, Fralick JA, Rolfe RD (2006) Genomic organization and molecular characterization of *Clostridium difficile* bacteriophage PhiCD119. J Bacteriol 188: 2568–2577. 16547044 10.1128/JB.188.7.2568-2577.2006 PMC1428422

[43] Casas V, Rohwer F (2007) Phage metagenomics. Methods Enzymol 421: 259–268. 17352928 10.1016/S0076-6879(06)21020-6

[44] Zheng ZL, Advani A, Melefors O, Glavas S, Nordstrom H, et al (2011) Titration-free 454 sequencing using Y adapters. Nature Protocols 6: 1367–1376. 21886102 10.1038/nprot.2011.369

[45] de la Bastide M, McCombie WR (2007) Assembling genomic DNA sequences with PHRAP. Curr Protoc Bioinformatics Chapter 11: Unit11 14. 10.1002/0471250953.bi1104s17 18428783

[46] Rosseel T, Scheuch M, Hoper D, De Regge N, Caij AB, et al (2012) DNase SISPA-next generation sequencing confirms Schmallenberg virus in Belgian field samples and identifies genetic variation in Europe. PLoS One 7: e41967. 22848676 10.1371/journal.pone.0041967 PMC3407049

[47] Carver T, Thomson N, Bleasby A, Berriman M, Parkhill J (2009) DNAPlotter: circular and linear interactive genome visualization. Bioinformatics 25: 119–120. 18990721 10.1093/bioinformatics/btn578 PMC2612626

[48] Schattner P, Brooks AN, Lowe TM (2005) The tRNAscan-SE, snoscan and snoGPS web servers for the detection of tRNAs and snoRNAs. Nucleic Acids Res 33: W686–689. 15980563 10.1093/nar/gki366 PMC1160127

[49] Besemer J, Borodovsky M (1999) Heuristic approach to deriving models for gene finding. Nucleic Acids Res 27: 3911–3920. 10481031 10.1093/nar/27.19.3911 PMC148655

[50] Altschul SF, Madden TL, Schaffer AA, Zhang J, Zhang Z, et al (1997) Gapped BLAST and PSI-BLAST: a new generation of protein database search programs. Nucleic Acids Res 25: 3389–3402. 9254694 10.1093/nar/25.17.3389 PMC146917

[51] Reese MG (2001) Application of a time-delay neural network to promoter annotation in the *Drosophila melanogaster* genome. Comput Chem 26: 51–56. 11765852 10.1016/s0097-8485(01)00099-7

[52] Moak M, Molineux IJ (2004) Peptidoglycan hydrolytic activities associated with bacteriophage virions. Mol Microbiol 51: 1169–1183. 14763988 10.1046/j.1365-2958.2003.03894.x

[53] Welker M, Erhard M (2007) Consistency between chemotyping of single filaments of *Planktothrix rubescens* (cyanobacteria) by MALDI-TOF and the peptide patterns of strains determined by HPLC-MS. Journal of Mass Spectrometry 42: 1062–1068. 17605146 10.1002/jms.1237

[54] Morello E, Saussereau E, Maura D, Huerre M, Touqui L, et al (2011) Pulmonary bacteriophage therapy on *Pseudomonas aeruginosa* cystic fibrosis strains: first steps towards treatment and prevention. PLoS One 6: e16963. 21347240 10.1371/journal.pone.0016963 PMC3039662

[55] Krumsiek J, Arnold R, Rattei T (2007) Gepard: a rapid and sensitive tool for creating dotplots on genome scale. Bioinformatics 23: 1026–1028. 17309896 10.1093/bioinformatics/btm039

[56] Carver T, Berriman M, Tivey A, Patel C, Bohme U, et al (2008) Artemis and ACT: viewing, annotating and comparing sequences stored in a relational database. Bioinformatics 24: 2672–2676. 18845581 10.1093/bioinformatics/btn529 PMC2606163

[57] Chenna R, Sugawara H, Koike T, Lopez R, Gibson TJ, et al (2003) Multiple sequence alignment with the Clustal series of programs. Nucleic Acids Res 31: 3497–3500. 12824352 10.1093/nar/gkg500 PMC168907

[58] Tamura K, Peterson D, Peterson N, Stecher G, Nei M, et al (2011) MEGA5: molecular evolutionary genetics analysis using maximum likelihood, evolutionary distance, and maximum parsimony methods. Mol Biol Evol 28: 2731–2739. 21546353 10.1093/molbev/msr121 PMC3203626

[59] Som A, Fuellen G (2009) The effect of heterotachy in multigene analysis using the neighbor joining method. Mol Phylogenet Evol 52: 846–851. 19482090 10.1016/j.ympev.2009.05.025

[60] Zafar N, Mazumder R, Seto D (2002) CoreGenes: a computational tool for identifying and cataloging “core” genes in a set of small genomes. BMC Bioinformatics 3: 12. 11972896 10.1186/1471-2105-3-12 PMC111185

[61] Mahadevan P, King JF, Seto D (2009) Data mining pathogen genomes using GeneOrder and CoreGenes and CGUG: gene order, synteny and in silico proteomes. Int J Comput Biol Drug Des 2: 100–114. 20054988 10.1504/ijcbdd.2009.027586

[62] Klumpp J, Dorscht J, Lurz R, Bielmann R, Wieland M, et al (2008) The terminally redundant, nonpermuted genome of Listeria bacteriophage A511: a model for the SPO1-like myoviruses of gram-positive bacteria. J Bacteriol 190: 5753–5765. 18567664 10.1128/JB.00461-08 PMC2519532

[63] Just W, Klotz G (1990) Terminal redundancy and circular permutation of mycoplasma virus L3 DNA. J Gen Virol 71 (Pt 9): 2157–2162. 10.1099/0022-1317-71-9-2157 2212995

[64] Alemayehu D, Casey PG, McAuliffe O, Guinane CM, Martin JG, et al (2012) Bacteriophages phiMR299-2 and phiNH-4 can eliminate *Pseudomonas aeruginosa* in the murine lung and on cystic fibrosis lung airway cells. MBio 3: e00029–00012. 22396480 10.1128/mBio.00029-12 PMC3302570

[65] Ceyssens PJ, Hertveldt K, Ackermann HW, Noben JP, Demeke M, et al (2008) The intron-containing genome of the lytic *Pseudomonas* phage LUZ24 resembles the temperate phage PaP3. Virology 377: 233–238. 18519145 10.1016/j.virol.2008.04.038

[66] Hertveldt K, Lavigne R, Pleteneva E, Sernova N, Kurochkina L, et al (2005) Genome comparison of *Pseudomonas aeruginosa* large phages. J Mol Biol 354: 536–545. 16256135 10.1016/j.jmb.2005.08.075

