# Supplementary material for: Genomic and Proteomic Analyses of the Terminally Redundant Genome of the Pseudomonas aeruginosa Phage PaP1: Establishment of Genus PaP1-Like Phages
Source: PLoS One. 2013 May 13;8(5):e62933. doi: 10.1371/journal.pone.0062933 (PMC3652863; doi:10.1371/journal.pone.0062933)
Supplement: Table S1 — Predicted genes and proteins of phage PaP1. (DOCX) [file pone.0062933.s001.docx]

**Table S1.** Predicted genes and proteins of phage PaP1.

| **Gene** | **Strand** | **From** | **To** | **Length**  **(aa)** | **Mass**  **(kDa)** | **Most significant matches [Corresponding species]** | **E-Value** | **Identity** |
| --- | --- | --- | --- | --- | --- | --- | --- | --- |
| 1 | + | 287 | 445 | 52 | 5.29 | No significant similarity found |  |  |
| 2 | - | 499 | 765 | 88 | 10.56 | No significant similarity found |  |  |
| 3 | - | 758 | 1168 | 136 | 15.5 | hypothetical protein ORF_0003 [*Pseudomonas* phage PAK_P1] | 4.00E^-46^ | 61% |
| 4 | - | 1158 | 1460 | 100 | 11.7 | hypothetical protein ORF_0004 [*Pseudomonas* phage PAK_P1] | 4.00E^-65^ | 87% |
| 5 | - | 1597 | 2283 | 228 | 27.8 | hypothetical protein ORF_0005 [*Pseudomonas* phage PAK_P1] | 8.00E^-101^ | 84% |
| 6 | - | 2286 | 2564 | 92 | 10.51 | No significant similarity found |  |  |
| 7 | - | 2552 | 2863 | 103 | 11.99 | hypothetical protein ORF_0006 [*Pseudomonas* phage PAK_P1] | 9.00E^-53^ | 100% |
| 8 | - | 2863 | 3225 | 120 | 13.82 | hypothetical protein ORF_0007 [*Pseudomonas* phage PAK_P1] | 1.00E^-52^ | 82% |
| 9 | - | 3215 | 3499 | 94 | 11.43 | hypothetical protein PP-LIT1_gp06 [*Pseudomonas* phage LIT1] | 2.00E^-10^ | 41% |
| 10 | - | 3767 | 4141 | 124 | 14.02 | hypothetical protein ORF_0009 [*Pseudomonas* phage PAK_P1] | 2.00E^-65^ | 96% |
| 11 | - | 4110 | 4679 | 189 | 21.99 | hypothetical protein ORF_0010 [*Pseudomonas* phage PAK_P1] | 2.00E^-93^ | 99% |
| 12 | - | 4600 | 4977 | 125 | 14.9 | Predicted DNA methylase [*Cyanothece* sp. CCY0110] | 3.2 | 30% |
| 13 | - | 4997 | 5578 | 193 | 21.52 | conserved hypothetical protein [*Aeromonas* phage PX29] | 1.00E^-18^ | 35% |
| 14 | - | 5575 | 5892 | 105 | 12.08 | hypothetical protein ORF_0013 [*Pseudomonas* phage PAK_P1] | 2.00E^-53^ | 98% |
| 15 | - | 5894 | 6043 | 49 | 5.76 | hypothetical protein ORF_0014 [*Pseudomonas* phage PAK_P1] | 6.00E^-19^ | 94% |
| 16 | - | 6055 | 6534 | 159 | 17.77 | conserved hypothetical protein [*Acinetobacter* phage 133] | 1.00E^-21^ | 42% |
| 17 | - | 6531 | 6785 | 84 | 10.03 | hypothetical protein ORF_0016 [*Pseudomonas* phage PAK_P1] | 1.00E^-22^ | 79% |
| 18 | - | 6742 | 8430 | 562 | 62.91 | NPT family protein [*Citrobacter youngae* ATCC 29220] | 1.00E^-121^ | 46% |
| 19 | - | 8487 | 8594 | 35 | 4.3 | hypothetical protein ORF_0018 [*Pseudomonas* phage PAK_P1] | 2.00E^-29^ | 90% |
| 20 | - | 8702 | 9568 | 288 | 31.8 | PRP synthetase [*Achromobacter piechaudii* ATCC 43553] | 4.00E^-41^ | 40% |
| 21 | - | 9578 | 9862 | 94 | 10.91 | putative ATPase [*Leptospira biflexa* serovar Patoc strain 'Patoc 1] | 8.00E^-18^ | 39% |
| 22 | - | 10005 | 10922 | 305 | 34.96 | RNA ligase 1 and tail attachment protein [*Escherichia* phage rv5] | 3.00E^-44^ | 40% |
| 23 | - | 10934 | 11341 | 135 | 15.22 | hypothetical protein ORF_0023 [*Pseudomonas* phage PAK_P1] | 2.00E^-54^ | 73% |
| 24 | - | 11338 | 11649 | 103 | 11.77 | hypothetical protein ORF_0024 [*Pseudomonas* phage PAK_P1] | 9.00E^-42^ | 90% |
| 25 | - | 11615 | 11872 | 85 | 9.53 | hypothetical protein ORF_0025 [*Pseudomonas* phage PAK_P1] | 2.00E^-31^ | 85% |
| 26 | - | 11853 | 12419 | 188 | 22.14 | putative phosphoesterase [*Aurantimonas manganoxydans* SI85-9A1] | 6.00E^-27^ | 40% |
| 27 | - | 12419 | 12850 | 143 | 17.18 | hypothetical protein ORF_0027 [*Pseudomonas* phage PAK_P1] | 1.00E^-77^ | 99% |
| 28 | - | 12840 | 13400 | 186 | 21.14 | MDP, HD region [*Lentisphaera araneosa* HTCC2155] | 6.00E^-10^ | 33% |
| 29 | - | 13402 | 13962 | 186 | 21.14 | putative cell wall hydrolase [*Pseudomonas* phage KPP10] | 2.00E^-43^ | 48% |
| 30 | - | 14020 | 14484 | 154 | 17.3 | hypothetical protein ORF_0031 [*Pseudomonas* phage PAK_P1] | 1.00E^-82^ | 96% |
| 31 | - | 14497 | 15576 | 359 | 41.26 | DNA ligase [*Pseudomonas* phage PAK_P1] | 0 | 97% |
| 32 | - | 15702 | 16091 | 129 | 14.35 | putative dCMP deaminase [*Pseudomonas* phage KPP10] | 4.00E^-33^ | 60% |
| 33 | - | 16121 | 16357 | 78 | 8.93 | hypothetical protein ORF_0035 [*Pseudomonas* phage PAK_P1] | 6.00E^-28^ | 86% |
| 34 | - | 16367 | 16579 | 70 | 7.45 | hypothetical protein ORF_0036 [*Pseudomonas* phage PAK_P1] | 3.00E^-32^ | 100% |
| 35 | - | 16576 | 16845 | 89 | 10 | hypothetical protein [*Pseudomonas* phage KPP10] | 1.00E^-09^ | 44% |
| 36 | - | 16854 | 17171 | 105 | 11.99 | hypothetical protein ORF_0037 [*Pseudomonas* phage PAK_P1] | 7.00E^-61^ | 98% |
| 37 | - | 17185 | 17463 | 92 | 10.47 | hypothetical protein ORF_0038 [*Pseudomonas* phage PAK_P1] | 1.00E^-36^ | 85% |
| 38 | - | 17773 | 17958 | 61 | 7.12 | hypothetical protein ORF_0040 [*Pseudomonas* phage PAK_P1] | 2.00E^-27^ | 100% |
| 39 | - | 17959 | 18183 | 74 | 8.53 | hypothetical protein ORF_0041 [*Pseudomonas* phage PAK_P1] | 1.00E^-36^ | 99% |
| 40 | - | 18180 | 18365 | 61 | 7.13 | hypothetical protein ORF_0042 [*Pseudomonas* phage PAK_P1] | 4.00E^-25^ | 96% |
| 41 | - | 18426 | 18974 | 182 | 20.41 | ClpP ATP-dependent protease subunit [*Escherichia* phage rv5] | 1.00E^-09^ | 33% |
| 42 | - | 19022 | 19378 | 118 | 13.08 | hypothetical protein ORF_0044 [*Pseudomonas* phage PAK_P1] | 6.00E^-59^ | 99% |
| 43 | - | 19375 | 19842 | 155 | 18.04 | aminotransferase [*Maritimibacter alkaliphilus* HTCC2654] | 0.062 | 30% |
| 44 | + | 20304 | 20492 | 62 | 6.93 | No significant similarity found |  |  |
| 45^a^ | + | 20635 | 20958 | 107 | 12.06 | hypothetical protein ORF_0047 [*Pseudomonas* phage PAK_P1] | 2.00E^-54^ | 99% |
| 46^a^ | + | 23542 | 25062 | 506 | 57.09 | putative large terminase subunit [*Pseudomonas* phage PAK_P1] | 0 | 100% |
| 47 | + | 25075 | 26514 | 479 | 54.23 | Phage conserved protein [*Enterobacteria* phage Felix 01] | 9.00E^-102^ | 40% |
| 48 | + | 26524 | 26994 | 156 | 17.19 | DNA methyltransferase [*Haliangium ochraceum* DSM 14365] | 1.00E^-09^ | 33% |
| 49 | + | 26991 | 27908 | 305 | 33.07 | hypothetical protein ORF_0052 [*Pseudomonas* phage PAK_P1] | 3.00E^-172^ | 99% |
| 50 | + | 27936 | 28346 | 136 | 14.87 | hypothetical protein ORF_0053 [*Pseudomonas* phage PAK_P1] | 4.00E^-73^ | 100% |
| 51 | + | 28390 | 29424 | 344 | 39.38 | major capsid protein [*Pseudomonas* phage PAK_P1] | 0 | 100% |
| 52 | + | 29474 | 29950 | 158 | 18.13 | hypothetical protein ORF_0055 [*Pseudomonas* phage PAK_P1] | 7.00E^-88^ | 100% |
| 53 | + | 30129 | 30401 | 90 | 10.47 | putative RNA polymerase [*Pseudomonas* phage KPP10] | 3.00E^-45^ | 59% |
| 54 | + | 30401 | 30781 | 126 | 14.35 | hypothetical protein ORF_0057 [*Pseudomonas* phage PAK_P1] | 4.00E^-69^ | 99% |
| 55 | + | 30778 | 31341 | 187 | 21.29 | cysteine methyltransferase [*Geobacillus* sp. G11MC16] | 0.28 | 31% |
| 56 | + | 31354 | 32640 | 428 | 46.37 | putative structural protein [*Pseudomonas* phage KPP10] | 2.00E^-112^ | 50% |
| 57 | + | 32671 | 33195 | 174 | 18.97 | putative structural protein [*Pseudomonas* phage KPP10] | 2.00E^-47^ | 58% |
| 58 | + | 33399 | 33776 | 125 | 13.7 | putative structural protein [*Pseudomonas* phage KPP10] | 5.00E^-43^ | 51% |
| 59 | + | 33769 | 34248 | 159 | 17.7 | putative structural protein [*Pseudomonas* phage KPP10] | 1.00E^-22^ | 41% |
| 60 | + | 34262 | 34633 | 123 | 13.62 | putative structural protein [*Pseudomonas* phage KPP10] | 3.00E^-16^ | 39% |
| 61 | + | 34890 | 37256 | 788 | 85.87 | putative tape measure protein [*Pseudomonas* phage KPP10] | 4.00E^-123^ | 44% |
| 62 | + | 37253 | 38014 | 253 | 28.55 | hypothetical protein ORF_0066 [*Pseudomonas* phage PAK_P1] | 1.00E^-139^ | 95% |
| 63 | + | 38020 | 38376 | 118 | 13.98 | hypothetical protein ORF_0068 [*Pseudomonas* phage PAK_P1] | 6.00E^-61^ | 95% |
| 64 | + | 38631 | 39290 | 219 | 24.16 | hypothetical protein ORF_0069 [*Pseudomonas* phage PAK_P1] | 2.00E^-172^ | 97% |
| 65 | + | 39287 | 40027 | 246 | 26.69 | putative baseplate protein [*Pseudomonas* phage KPP10] | 3.00E^-64^ | 56% |
| 66 | + | 40039 | 40410 | 123 | 14.18 | hypothetical protein ORF_0071 [*Pseudomonas* phage PAK_P1] | 7.00E^-64^ | 96% |
| 67 | + | 40412 | 41875 | 487 | 52.4 | putative base plate related protein [*Pseudomonas* phage KPP10] | 1.00E^-100^ | 45% |
| 68 | + | 41894 | 42625 | 243 | 26.68 | hypothetical protein ORF_0073 [*Pseudomonas* phage PAK_P1] | 2.00E^-132^ | 97% |
| 69 | + | 42636 | 44648 | 670 | 69.79 | putative tail fiber protein [*Pseudomonas* phage KPP10] | 0 | 65% |
| 70 | + | 44715 | 45062 | 115 | 13.56 | hypothetical protein ORF_0075 [*Pseudomonas* phage PAK_P1] | 5.00E^-55^ | 81% |
| 71 | + | 45076 | 46575 | 499 | 53.09 | putative tail fiber protein [*Pseudomonas* phage KPP10] | 3.00E^-113^ | 61% |
| 72 | + | 46592 | 47152 | 186 | 20.92 | endolysin [*Pseudomonas* phage PaP1] | 3.00E^-106^ | 100% |
| 73 | + | 47170 | 47409 | 79 | 8.5 | hypothetical protein ORF_0078 [*Pseudomonas* phage PAK_P1] | 4.00E^-35^ | 95% |
| 74 | + | 47396 | 47839 | 147 | 15.92 | hypothetical protein ORF_0079 [*Pseudomonas* phage PAK_P1] | 2.00E^-80^ | 99% |
| 75 | + | 47971 | 48276 | 101 | 11.47 | hypothetical protein ORF_0080 [*Pseudomonas* phage PAK_P1] | 1.00E^-49^ | 99% |
| 76 | - | 48664 | 48975 | 103 | 11.61 | hypothetical protein ORF_0081 [*Pseudomonas* phage PAK_P1] | 3.00E^-36^ | 72% |
| 77 | - | 49309 | 50118 | 269 | 30.4 | hypothetical protein ORF_0082 [*Pseudomonas* phage PAK_P1] | 6.00E^-134^ | 88% |
| 78 | - | 50111 | 50269 | 52 | 5.8 | hypothetical protein ORF_0083 [*Pseudomonas* phage PAK_P1] | 8.00E^-07^ | 53% |
| 79 | - | 50272 | 51408 | 378 | 42.6 | RNA ligase, DRB0094 family [*Chitinophaga pinensis* DSM 2588] | 4.00E^-53^ | 37% |
| 80 | + | 52124 | 52321 | 65 | 7.45 | hypothetical protein ORF_0087 [*Pseudomonas* phage PAK_P1] | 3.00E^-27^ | 93% |
| 81 | + | 52408 | 52809 | 133 | 15.77 | hypothetical protein ORF_0088 [*Pseudomonas* phage PAK_P1] | 4.00E^-96^ | 99% |
| 82 | + | 52946 | 53488 | 180 | 20.48 | hypothetical protein ORF_0090 [*Pseudomonas* phage PAK_P1] | 3.00E^-67^ | 95% |
| 83 | + | 53485 | 54141 | 218 | 24.17 | hypothetical protein ORF_0091 [*Pseudomonas* phage PAK_P1] | 2.00E^-111^ | 95% |
| 84 | + | 54128 | 54292 | 54 | 6.43 | hypothetical protein ORF_0092 [*Pseudomonas* phage PAK_P1] | 2.00E^-23^ | 100% |
| 85 | + | 54295 | 54597 | 100 | 11.9 | hypothetical protein ORF_0093 [*Pseudomonas* phage PAK_P1] | 9.00E^-52^ | 99% |
| 86 | + | 54712 | 55020 | 102 | 12.03 | hypothetical protein ORF_0094 [*Pseudomonas* phage PAK_P1] | 7.00E^-79^ | 99% |
| 87 | + | 55253 | 55444 | 63 | 7.12 | hypothetical protein ORF_0096 [*Pseudomonas* phage PAK_P1] | 7.00E^-27^ | 94% |
| 88 | + | 55696 | 55881 | 61 | 7.19 | No significant similarity found |  |  |
| 89 | + | 55935 | 57797 | 620 | 70.57 | primase/helicase [*Pseudomonas* phage PAK_P1] | 0 | 99% |
| 90 | + | 57858 | 60572 | 904 | 103.35 | DNA polymerase [*Pseudomonas* phage PAK_P1] | 0 | 99% |
| 91 | + | 60664 | 61062 | 132 | 14.37 | hypothetical protein ORF_0099 [*Pseudomonas* phage PAK_P1] | 2.00E^-67^ | 97% |
| 92 | + | 61091 | 61258 | 55 | 6.15 | hypothetical protein ORF_0100 [*Pseudomonas* phage PAK_P1] | 2.00E^-23^ | 99% |
| 93 | + | 61260 | 61970 | 236 | 26.27 | hypothetical protein ORF_0101 [*Pseudomonas* phage PAK_P1] | 2.00E^-113^ | 84% |
| 94 | + | 62072 | 63076 | 334 | 37.12 | hypothetical protein ORF_0102 [*Pseudomonas* phage PAK_P1] | 0 | 97% |
| 95 | + | 63146 | 63379 | 77 | 8.39 | hypothetical protein ORF_0104 [*Pseudomonas* phage PAK_P1] | 2.00E^-23^ | 72% |
| 96 | + | 63389 | 63610 | 73 | 8.22 | hypothetical protein ORF_0105 [*Pseudomonas* phage PAK_P1] | 3.00E^-27^ | 80% |
| 97 | + | 63652 | 64704 | 350 | 39.97 | putative exodeoxyribonuclease [*Pseudomonas* phage KPP10] | 3.00E^-62^ | 39% |
| 98 | + | 64701 | 65264 | 187 | 21.57 | phage protein [*Enterobacteria* phage phiEcoM-GJ1] | 1.00E^-19^ | 36% |
| 99 | + | 65261 | 65659 | 132 | 15.19 | hypothetical protein ORF_0108 [*Pseudomonas* phage PAK_P1] | 1.00E^-71^ | 99% |
| 100 | + | 65680 | 65886 | 68 | 7.56 | hypothetical protein ORF_0109 [*Pseudomonas* phage PAK_P1] | 2.00E^-24^ | 65% |
| 101 | + | 65859 | 66320 | 153 | 17.31 | hypothetical protein ORF_0110 [*Pseudomonas* phage PAK_P1] | 2.00E^-63^ | 83% |
| 102 | + | 66317 | 66484 | 55 | 6.49 | hypothetical protein ORF_0111 [*Pseudomonas* phage PAK_P1] | 7.00E^-22^ | 93% |
| 103 | + | 66429 | 66668 | 79 | 9.29 | No significant similarity found |  |  |
| 104 | + | 66650 | 67444 | 264 | 29.81 | constituent protein [*Pseudomonas* phage PaP3] | 3.00E^-43^ | 42% |
| 105 | + | 67441 | 67623 | 60 | 6.97 | No significant similarity found |  |  |
| 106 | + | 67643 | 67852 | 69 | 7.5 | hypothetical protein ORF_0113 [*Pseudomonas* phage PAK_P1] | 2.00E^-31^ | 100% |
| 107 | + | 67872 | 68207 | 111 | 12.5 | hypothetical protein ORF_0114 [*Pseudomonas* phage PAK_P1] | 7.00E^-57^ | 97% |
| 108 | + | 68259 | 68426 | 55 | 6.13 | hypothetical protein ORF_0115 [*Pseudomonas* phage PAK_P1] | 2.00E^-26^ | 89% |
| 109 | + | 68581 | 69369 | 262 | 29.36 | gp41 [Enterobacteria phage N4] | 1.00E^-13^ | 54% |
| 110 | + | 69576 | 70538 | 320 | 36.88 | thymidylate synthase [*Escherichia* phage rv5] | 4.00E^-79^ | 49% |
| 111 | + | 70495 | 70884 | 129 | 15.04 | hypothetical protein ORF_0118 [*Pseudomonas* phage PAK_P1] | 2.00E^-62^ | 100% |
| 112 | + | 70901 | 71947 | 348 | 40.34 | RDR beta subunit [*Pseudomonas* phage PAK_P1] | 0 | 99% |
| 113 | + | 71940 | 73685 | 581 | 66.47 | RDR alpha chain [*Pseudomonas* phage PAK_P1] | 0 | 99% |
| 114 | + | 73834 | 74136 | 100 | 11.71 | hypothetical protein ORF_0121 [*Pseudomonas* phage PAK_P1] | 3.00E^-51^ | 97% |
| 115 | + | 74136 | 74357 | 73 | 8.33 | No significant similarity found |  |  |
| 116 | + | 74370 | 74609 | 79 | 9.21 | hypothetical protein ORF_0122 [*Pseudomonas* phage PAK_P1] | 5.00E^-38^ | 100% |
| 117 | + | 74606 | 74881 | 91 | 10.45 | hypothetical protein ORF_0123 [*Pseudomonas* phage PAK_P1] | 6.00E^-45^ | 94% |
| 118 | + | 74883 | 75260 | 125 | 13.75 | hypothetical protein ORF_0124 [*Pseudomonas* phage PAK_P1] | 1.00E^-30^ | 60% |
| 119 | + | 75266 | 75451 | 61 | 6.97 | hypothetical protein ORF_0125 [*Pseudomonas* phage PAK_P1] | 7.00E^-28^ | 99% |
| 120 | + | 75497 | 75736 | 79 | 9.06 | hypothetical protein ORF_0126 [*Pseudomonas* phage PAK_P1] | 3.00E^-38^ | 99% |
| 121 | + | 75756 | 76259 | 167 | 18.4 | hypothetical protein ORF_0127 [*Pseudomonas* phage PAK_P1] | 4.00E^-67^ | 85% |
| 122 | + | 76269 | 76463 | 64 | 7.18 | hypothetical protein ORF_0128 [*Pseudomonas* phage PAK_P1] | 3.00E^-25^ | 88% |
| 123 | + | 76465 | 76695 | 76 | 8.68 | hypothetical protein ORF_0129 [*Pseudomonas* phage PAK_P1] | 7.00E^-37^ | 100% |
| 124 | + | 76717 | 76968 | 83 | 10.1 | hypothetical protein ORF_0130 [*Pseudomonas* phage PAK_P1] | 6.00E^-41^ | 100% |
| 125 | + | 77127 | 78113 | 328 | 37.49 | hypothetical protein ORF_0131 [*Pseudomonas* phage PAK_P1] | 1.00E^-177^ | 95% |
| 126 | + | 79140 | 79616 | 158 | 18.19 | hypothetical protein ORF_0133 [*Pseudomonas* phage PAK_P1] | 2.00E^-66^ | 78% |
| 127 | + | 79692 | 79958 | 88 | 10.31 | hypothetical protein ORF_0134 [*Pseudomonas* phage PAK_P1] | 4.00E^-41^ | 92% |
| 128 | + | 79971 | 80114 | 47 | 5.41 | hypothetical protein ORF_0135 [*Pseudomonas* phage PAK_P1] | 1.00E^-18^ | 94% |
| 129 | + | 80114 | 80401 | 95 | 10.58 | hypothetical protein ORF_0136 [*Pseudomonas* phage PAK_P1] | 2.00E^-47^ | 99% |
| 130 | + | 80302 | 80646 | 114 | 12.49 | hypothetical protein [*Pseudomonas* phage KPP10] | 7.00E^-33^ | 89% |
| 131 | + | 80717 | 80845 | 42 | 4.87 | hypothetical protein ORF_0137 [*Pseudomonas* phage PAK_P1] | 9.00E^-15^ | 98% |
| 132 | + | 80845 | 81153 | 102 | 11.41 | hypothetical protein ORF_0138 [*Pseudomonas* phage PAK_P1] | 5.00E^-53^ | 99% |
| 133 | + | 81184 | 81375 | 63 | 7.28 | hypothetical protein ORF_0139 [*Pseudomonas* phage PAK_P1] | 8.00E^-16^ | 96% |
| 134 | + | 81612 | 81995 | 127 | 15.15 | hypothetical protein ORF_0140 [*Pseudomonas* phage PAK_P1] | 3.00E^-54^ | 82% |
| 135 | + | 82071 | 82742 | 223 | 24.38 | conserved hypothetical protein [Cyanophage Syn26] | 1.00E^-38^ | 41% |
| 136 | + | 83013 | 83438 | 141 | 15.57 | hypothetical protein ORF_0142 [*Pseudomonas* phage PAK_P1] | 5.00E^-69^ | 93% |
| 137 | + | 83630 | 83902 | 90 | 10.37 | hypothetical protein ORF_0144 [*Pseudomonas* phage PAK_P1] | 8.00E^-06^ | 80% |
| 138 | + | 83950 | 84144 | 64 | 6.94 | hypothetical protein ORF_0144 [*Pseudomonas* phage PAK_P1] | 2.00E^-27^ | 94% |
| 139 | + | 84162 | 84488 | 108 | 12.08 | hypothetical protein ORF_0145 [*Pseudomonas* phage PAK_P1] | 1.00E^-14^ | 45% |
| 140 | + | 84485 | 84709 | 74 | 8.45 | hypothetical protein ORF_0146 [*Pseudomonas* phage PAK_P1] | 7.00E^-34^ | 94% |
| 141 | + | 84742 | 85008 | 88 | 10.02 | hypothetical protein ORF_0147 [*Pseudomonas* phage PAK_P1] | 2.00E^-43^ | 97% |
| 142 | + | 85005 | 85397 | 130 | 15.07 | hypothetical protein ORF_0148 [*Pseudomonas* phage PAK_P1] | 8.00E^-63^ | 87% |
| 143 | + | 85512 | 86093 | 193 | 21.52 | hypothetical protein ORF_0149 [*Pseudomonas* phage PAK_P1] | 5.00E^-107^ | 98% |
| 144 | + | 86165 | 86410 | 81 | 9.14 | hypothetical protein ORF_0150 [*Pseudomonas* phage PAK_P1] | 6.00E^-40^ | 97% |
| 145 | + | 86422 | 86751 | 109 | 12.57 | hypothetical protein ORF_0151 [*Pseudomonas* phage PAK_P1] | 2.00E^-58^ | 100% |
| 146 | + | 86806 | 87288 | 160 | 17.9 | hypothetical protein ORF_0152 [*Pseudomonas* phage PAK_P1] | 7.00E^-91^ | 100% |
| 147 | + | 87375 | 87902 | 175 | 19.47 | hypothetical protein ORF_0153 [*Pseudomonas* phage PAK_P1] | 8.00E^-113^ | 98% |
| 148 | + | 87902 | 88183 | 93 | 10.31 | hypothetical protein ORF_0154 [*Pseudomonas* phage PAK_P1] | 1.00E^-45^ | 94% |
| 149 | + | 88211 | 88381 | 56 | 6.31 | No significant similarity found |  |  |
| 150 | + | 88436 | 88888 | 150 | 17.42 | hypothetical protein ORF_0155 [*Pseudomonas* phage PAK_P1] | 3.00E^-30^ | 51% |
| 151 | + | 88903 | 89133 | 76 | 8.8 | hypothetical protein PP-LIT1_gp03 [*Pseudomonas* phage LIT1] | 2.00E^-08^ | 42% |
| 152 | + | 89130 | 89417 | 95 | 11 | hypothetical protein ORF_0156 [*Pseudomonas* phage PAK_P1] | 5.00E^-33^ | 74% |
| 153 | + | 89501 | 89695 | 64 | 7.13 | hypothetical protein ORF_0158 [*Pseudomonas* phage PAK_P1] | 3.00E^-29^ | 100% |
| 154 | + | 89746 | 89883 | 45 | 4.77 | No significant similarity found |  |  |
| 155 | - | 89936 | 90067 | 43 | 4.72 | hypothetical protein [*Pseudomonas* phage KPP10] | 1.00E^-05^ | 62% |
| 156 | - | 90557 | 90694 | 45 | 5.02 | No significant similarity found |  |  |
| 157 | + | 90879 | 91481 | 200 | 22.82 | hypothetical protein ORF_0001 [*Pseudomonas* phage PAK_P1] | 1.00E^-112^ | 95% |

The listed data were acquired using BlastP against NCBI database: non-redundant protein sequences (nr). We mainly selected the proteins of PAK_P1 (with more detailed annotations) for the best matches.

NPT: nicotinamide phosphoribosyl transferase; PRP: phosphoribosylpyrophosphate; MDP: Metal dependent phosphohydrolase; RDR: ribonucleotide-diphosphate reductase.

^a^12 tRNA genes cluster in a region between gene 45 and 46.
